# Effect of Duodenogastric Reflux on Dental Enamel

**DOI:** 10.3290/j.ohpd.a45073

**Published:** 2020-09-04

**Authors:** Juliana Jendiroba Faraoni, Julia Barone de Andrade, Laís Lopes Machado de Matos, Regina Guenka Palma-Dibb

**Affiliations:** a Researcher, Department of Restorative Dentistry, School of Dentistry of Ribeirão Preto, University of São Paulo, Monte Alegre, Ribeirão Preto, SP, Brazil. Drafting the work; performance of the experimental section; writing the paper.; b Researcher, Department of Restorative Dentistry, School of Dentistry of Ribeirão Preto, University of São Paulo, Monte Alegre, Ribeirão Preto, SP, Brazil. Substantial contributions to the study design; the acquisition, analysis and master’s thesis.; c Doctoral Student, Department of Restorative Dentistry, School of Dentistry of Ribeirão Preto, University of São Paulo, Monte Alegre, Ribeirão Preto, SP, Brazil. Analysis and interpretation of data for the study.; d Full Professor, Department of Restorative Dentistry, School of Dentistry of Ribeirão Preto, University of São Paulo, Monte Alegre, Ribeirão Preto, SP, Brazil. Substantial contributions to the study design; analysis, interpretation of data for the study; supervisor of Master’s thesis.

**Keywords:** reflux, dental erosion, duodenogastric reflux, enamel

## Abstract

**Purpose:**

To evaluate the effects of stomach and duodenal fluid on enamel surfaces, simulating the action of refluxed liquid in patients with duodenogastric reflux.

**Methods and Materials:**

Forty bovine incisors were used to obtain enamel fragments. Only half of the enamel surface was exposed to erosive challenges; the samples were then randomly divided into the following four groups (n = 10): G1: HCl; G2: HCl + pepsin; G3: HCl + ox bile + NaHCO_3_; and G4: HCl + pancreatin + NaHCO_3_. The specimens were placed in 37°C solutions, six times per day, for 20 s, over a period of 5 days and then analysed for morphology, surface roughness and the step formed on the dental enamel using confocal laser microscopy. The data were analysed using the Kruskal-Wallis and Dunn’s test (p <0.05).

**Results:**

Both analyses revealed a higher step and surface roughness for the G3 group (5.6 μm ± 1.69, 2.2 μm ± 1.61), which were statistically significant compared with the G1 and G2 groups (3.9 μm ± 1.5 μm; 1.0 μm ± 0.18; 3.7 μm ± 1.45; and 0.9 μm ± 0.12) (p <0.05); only the step in the G4 group (4.9 μm ± 1.8 μm) was similar to that of the G3 group (p >0.05). Morphological analysis showed greater structural loss in the G3 and G4 groups.

**Conclusions:**

Bile and pancreatin, in combination with hydrochloric acid, may promote a greater loss of structure, increased surface roughness and loss of enamel prismatic anatomy.

Duodenum-gastric reflux is a normal physiological event in prolonged fasting periods. Its pathogenicity depends on the rhythm, amount and duration of gastric exposure to their contents.^12^

Duodenum-gastroesophageal reflux (DGER) is characterised by the contents from the stomach and duodenal origin being regurgitated into the oesophagus.^33^ The rise in pH was more commonly associated with pancreatic enzymes than with bile.^7^ One study identified bile acids in the saliva of 32.6% of patients who underwent bariatric surgery.^5^

Bile reflux gastritis occurs because of the excessive flow of bile, pancreatic juice and intestinal secretions into the stomach,^2^ and studies have shown that alone or in association with gastric juice, duodenal contents reflowing into the oesophagus can cause severe oesophagitis^4,23^ and increased gastric mucosal injury.^4^ Both bile acids and duodenal origin enzymes can cause injury to the gastrointestinal mucosa.^11^

Pepsin is an enzyme that is only produced in the stomach.^1^ Pepsin is more active at pH ranges from 2.0 to 3.0, and its activity decreases with a reduction in acidity.^25^ The same process occurs with duodenum enzymes, such as pancreatin and bile, both of which can be found in the saliva of patients with duodenal-gastric reflux.^27^ These enzymes can reach and harm the dental tissue and thus promote wear of dental surfaces.

The bile-acid mixture after duodenum-gastric reflux often has a final pH of 4.0 and 7.0. However in cases with more severe oesophagitis, this reflux can reach lower pHs (pH 2–4).^23^ The decreased acid reflux explains a statistically significant portion of cases that continue to have symptoms after conventional or acid reflux is treated.^19^

Dental erosion, which is an extraoesophageal symptom that is most prevalent for reflux in the oral cavity,^15^ can be defined as the loss of mineral material that has been chemically removed from the tooth surface due to acid and alkaline substances; such erosion is considered an irreversible and sterile phenomenon.^32^ Degradation of the tooth surface can occur due to factors of intrinsic origin, extrinsic origin or a combination of both. Intrinsic factors include gastroesophageal reflux disorder, duodenum-gastric reflux disorder, eating and physiology disorder and saliva alterations; extrinsic factors include dietary acid and professional occupation.^24^ Clinically, a loss of gloss enamel, wide and shallow lesions without a sharp angle, an occlusal surface without anatomy, incisal with increased translucency, prominent restorations, loss of enamel on the lingual face of incisors and canines are observed, all of which made early diagnosis difficult.^17^ With the advancement of wear, the lesions become visible and may reach the dentine, thereby causing hypersensitivity^21^ at this stage; a restorative procedure may be necessary.

Dental erosion is a common condition that increases with age.^14^ Apparently, the first stage of erosion caused by DGER is the loss of surface enamel caused by the regurgitation of liquid or gaseous hydrochloric acid; subsequently, the destruction of the dental element is accelerated through mixing the gastric and duodenal contents.^9^

According to the literature, both the substances with a gastric (HCl and pepsin) and duodenal origin (bile and pancreatic juice) can remove mineral from tooth surfaces and cause erosion lesions for the majority of individuals with duodenal-gastric reflux.

As such, the objective of this study was to evaluate the effects of the liquid content originating from the stomach and duodenum on the surface of bovine dental enamel, simulating what occurs in the oral cavity of patients with duodenum-gastric reflux disease.

## Methods and Materials

### Preparation and Selection of Dental Fragments

Bovine incisors were obtained from a slaughterhouse (Mondelli Food Industry, Bauru, São Paulo, Brazil). Bovine incisors freshly extracted and stored in a 0.1% thymol solution (pH = 7.0) at 4°C and then cleaned with a scaler and water/pumice slurry in dental prophylactic cups. Teeth were sectioned using a water-cooled diamond disk # 7015 (KG Sorensen, São Paulo, Brazil) in a sectioning machine (Isomet 1000, Buehler, Lake Bluff, USA) in order to obtain enamel slabs measuring 4 × 4 mm. Then, the enamel surfaces of the fragments were planed and polished with aluminium oxide sandpaper and a felt disc with a suspension of alumina.

The specimens were ground flat, finished and polished with #400, #600 and #1200 grit sandpaper and felt disks impregnated with alumina 0.3 μm and 0.05 μm (Arotec, Cotia, SP, Brazil) using a polishing machine (APL-4, Arotec S/A Ind. and Commerce, São Paulo/SP, Brazil). The experimental units were immersed in an ultrasonic bath with deionised water (Ultrasonic Cleaner T-1449-D, Odontobrás Indústria e Comércio, Ribeirão Preto, SP, Brazil) for 10 min, to remove polishing debris.

Then, the specimens without cracks, irregularities and imperfections were selected and subjected to an initial microhardness test (Shimadzu, Kyoto, Japan) with a static load of 25 g force applied for 10 s. Five indentations were made on each sample, and those that presented with a value 20% above or below the mean of all specimens and those with an internal standard deviation over 20% of the value of their own medium were discarded. At the end of this process, 40 specimens were selected.

Furthermore, the samples were covered with a composite resin (FiltekTM Z350, colour A1, 3M Oral Care, Monrovia, CA, USA) to protect all of its faces; only part of the external face (4 mm) was not covered with resin. This face was divided into two 2-mm parts, with one part of the enamel surface (control area) covered and the other exposed half being submitted to erosive challenges (≈ 8 mm^2^). The dental specimens were randomly (using software EXCEL, function aleatory, Microsoft, Redmond, WA, USA) divided into four experimental groups according to the substances to which they were exposed:

- Group1 – Exposure to HCl at 37°C (pH 2.0).
- Group 2 – Exposure to HCl + 0.1% pepsin at 37°C (pH 2.1).
- Group 3 – Exposure to HCl + 0.4% bile + 0.05%. NaHCO_3_ at 37°C (pH 3.0).
- Group 4 – Exposure to HCl + 0.1% pancreatin + 0.02% NaHCO_3_ at 37°C (pH 3.0).

### Preparation of Solutions

Hydrochloric acid solution – 0.82 ml of hydrochloric acid (ACS reagent at 37%; Sigma-Aldrich, Dorset, UK) – was diluted in 1 L of deionised water, obtaining a solution of 0.01 M HCl in pH 2.0 at 37°C.

Hydrochloric acid solution with pepsin: first, the hydrochloric acid solution was prepared and then 1 g of pepsin (pepsin lyophilised (salt-free) – Sigma-Aldrich) was diluted to obtain a solution of 0.01 M HCl and 0.1% pepsin with a final pH of 2.1 at 37°C.

Hydrochloric acid solution with bile and NaHCO_3_: hydrochloric acid solution was first prepared and then 4 g of bile bovine (Millipore, Burlington, MA, USA) and 0.5 g of NaHCO_3_ (Sigma-Aldrich) were diluted to obtain a solution of 0.01 M HCl, 0.4% pepsin and 0.05% of NaHCO_3_ with a final pH of 3.0 at 37°C. The pH of this solution was used for simulating cases with more severe duodenum-gastric reflux.

Hydrochloric acid solution with pancreatin and NaHCO_3_: first, the hydrochloric acid solution was prepared and then 1 g of pancreatin (taken from porcine pancreas – Sigma-Aldrich) and 0.2 g of NaHCO_3_ (Sigma-Aldrich) were diluted to obtain a solution of 0.01 M HCl, 0.1% pepsin and 0.02% of NaHCO_3_ with a final pH of 3.0 at 37°C.

### Erosion Protocol

Each specimen was immersed in 5 ml of the solution during 20 s of stirring at 200 rpm (mod. 752A, Fisatom Equipamentos Cientificos, Brazil). This process was performed six times per day for 5 days, with an interval of 1 h between each erosive challenge; in the interval between the challenges, the samples were kept immersed in 10 ml of artificial saliva.^22^ The solutions were prepared daily and kept at 37°C to maintain the action of the enzymes.

### Evaluation of the Step and Surface Roughness

After challenges were complete, the resin composite that covers the other half enamel surface was removed with scalpel blade carefully and analysis then performed. For the step (step of height formed after the erosion process with control surface/erosion surface) and roughness, the enamel specimens were evaluated using a confocal laser scanning microscope (CLSM) (Olympus LEXT, Japan) connected to a computer with specific software (LEXT, 3D Measuring Laser Microscope, software OLS 4000, Olympus, Tokyo, Japan). The 3D measuring laser microscope provides simultaneous acquisition of brightness, height and colour information in high resolution and high accuracy, using a diode laser of wavelength 405 µm and non-contact measurement. The images were collected with part control and part experimental area in 3D with a 5× objective that was later increased to 107×.

**Fig 1:**
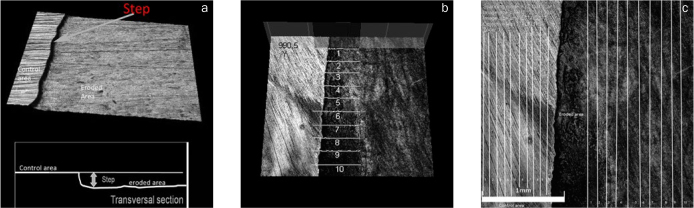
Illustrative images of CLSM for analysis of step and roughness. (a) 3D image of the step formed in the enamel surface after the erosion process. (b) Step measurements. (c) Roughness measurements (in the control area and eroded area).

The step was analysed based on the control area (protected), step height was defined as the distance between the base of the eroded region and height of the control region (Fig 1a). This was performed using 10-line profiles across the erosion/control interface, a mean and standard deviation of step height was then calculated for each sample (Fig 1b) to determine the level of wear using specific CLSM software (OLS 4000, Olympus).

For surface roughness, each specimen was filtered using the standard filtering in the OLS 4000 software (Olympus). Before measurements, the inclination was adjusted and a jagged correction filter was used prior to taking any data. For Ra, a cut-off λc of 80 µm was selected. Ten profiles in the parallel direction of interface control area/eroded were taken for each region (control and eroded) and then averaged to acquire the average Ras in µm (Fig 1c). For data analysis, the difference in the roughness of the experimental (Re) area was subtracted from the control area (Rc) in μm (Rf = Re – Rc).

### Morphological Evaluation

Morphologic analysis of enamel fragments was performed using CLSM (LEXT Olympus). The specimens were positioned parallel to a microscope that was connected to a computer with special software (OLS4000 Olympus) using a 100× objective lens with a numerical aperture of 0.95. Three-dimensional images were taken with an increased (2131×) objective, and morphological analysis was performed for all specimens.

### Data Analysis

Data for step and surface roughness were analysed for normality and homogeneity. These data were not found to be normal, and the non-parametric method of Kruskal–Wallis and Dunn’s test were used to evaluate the medians with α = 5% (TIBCO Software Statistica (data analysis software system)). The power test (α = 0.05) was 0.70 for step and 0.93 for roughness. For morphological analysis, a descriptive evaluation was performed.

## Results

In the step formed, there was a statistically significant difference among the groups (p <0.05); group 3 showed the highest value and was statistically significantly different from groups 1 and 2 (p <0.05), whereas group 4 was similar to all groups (p >0.05; Table 1).

**Table 1:** Mean (in µm) and standard deviation of the step measure of experimental groups (same letter indicates statistical similarity)

Groups	Step
G1 (HCl)	3.90 ± 1.56^b^
G2 (HCl + pepsin)	3.67 ± 1.45^b^
G3 (HCl + bile)	5.58 ± 1.70^a^
G4 (HCl + pancreatin)	4.90 ± 1.80^ab^

Analysing the roughness data, there were statistically significant differences among the groups (p <0.05); group 3 showed higher values and was statistically superior to the other groups (p <0.05). Groups 1, 2 and 4 were very similar to each other (p >0.05; Table 2).

**Table 2:** Mean (in µm) and the standard deviation of the surface roughness of experimental groups (same letter indicates statistical similarity)

Groups	Roughness values
G1 (HCl)	1.03 ± 0.18^b^
G2 (HCl + pepsin)	0.88 ± 0.23^b^
G3 (HCl + bile)	2.20 ± 1.61^a^
G4 (HCl + pancreatin)	0.93 ± 0.12^b^

**Fig 2:**
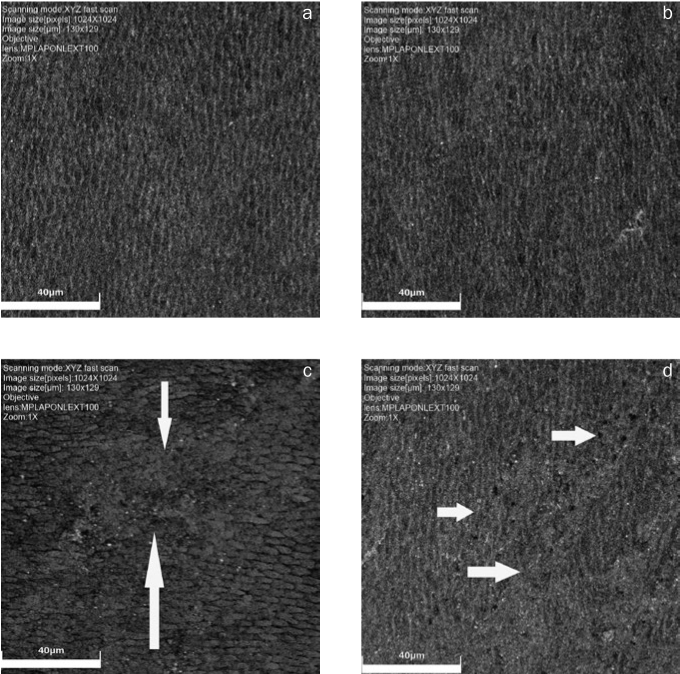
Illustrative images of the details of the enamel eroded by different solutions. Groups 1 (a) and 2 (b) have a more intense demineralisation process in the interprismatic region without destroying the head of the prisms. In groups 3 (c) and 4 (d), the solutions affected the enamel more strongly and destroyed the prism heads, resulting in amorphous enamel areas (arrows) (increase 2131×).

In the morphological analysis of the different groups (Fig 2), group 1 showed the lowest rung, and the good region interprismatic eroded, exposing the heads of the prisms. Group 2 also showed a smaller step, but it was more pronounced than group 1, and it had the most exposed enamel rods and structure loss areas. In groups 3 and 4, there was a major structural loss of the exposed enamel with intense removal of the interprismatic region and amorphous areas.

## Discussion

Gastroesophageal reflux disease (GERD) is considered a determining factor for the presence of dental erosion. Several studies have demonstrated such a correlation^6,26^ without a full understanding of the process. In some cases, dental erosion is the most obvious clinical sign of GERD, and a dentist may be the first professional to suspect the disease.^18^

Both gastric and duodenal-gastric reflux often occur in healthy patients and in patients with DGER.^10^ However, in the literature^3^ there are reports of a considerably higher concentration of bile in the stomach of patients with DGER (26.4 mmol/L) compared with healthy patients (4.45 mmol/L). In the saliva of patients undergoing bariatric surgery, the concentration of bile acids is higher,^5^ which could affect the oral tissues. In this way, material with a duodenal origin (bile and pancreatic) may be present in the oral cavity in both healthy people and patients with DGER.^27^ However, little is known about the effect of these substances on the enamel because no study has evaluated this substance in dental wear; therefore, the present study determined the effect of bile, pancreatin and pepsin on enamel.

The substrate employed in this study was a bovine tooth. This compound is widely used for studies that analyse the erosion process in enamel and has demonstrated good reproducibility and is comparable with human teeth. In addition, the enamel surface was flattened and polished, removing the irregular characteristic of the bovine enamel layer.^22^ Further, these animals come from slaughterhouses and are of the same age, eat the same type of food and have similar habits, facilitating standardisation.

It can be observed that the different solutions tested had varying behaviours, with the pancreatin and bile solutions causing more intense erosion. The step and surface roughness presented results that confirm those findings obtained in the qualitative analysis; both were higher in groups 3, followed by group 4, demonstrating increased destruction of dental fragments for the groups containing duodenal enzymes. A similar result was observed by Higo et al (2009),^9^ who observed the destruction of more accelerated dental elements after the induction of duodenal reflux in rats, a process which primarily occurred when there was a mixture of gastric and duodenal contents.^9^ Due to the absence of other relevant studies, it was not possible to correlate the occurrence of dental erosion with the solutions analysed in this study.

However, the solution with both pancreatin and bile was the most viscous, and after washing at the end of each challenge, some residue remained on the surface, likely intensifying the corrosive process. The methodology was not designed to intensely wash the surface because such an approach could increase the effect of the erosive process by fluid movement, which may have allowed for the accumulation of the substances, thereby generating a larger surface degradation.

Different levels of dental erosion can primarily be explained by the acid exposure time and frequency,^30^ with this study simulating mild reflux. Statistically significant loss of tooth structure was related to a high index of DGER^16,24^; however, this study had the objective of initially assessing the effects of different substances.

For dental enamel to be reached, the duodenal contents pass through the stomach into the oesophagus until they finally reach the oral cavity. Hydrochloric acid has substantial potential to erode enamel^31^ because of the loss of mineral acid into the medium.^28^ Then, there is an increase in the surface roughness^22^ followed by a reduction of the hardness.^29^ Consequently, HCl was maintained in all solutions; however, pH 3.0 was required to simulate most cases of DGER disease in the acidic environment with an oesophageal pH of <4.29.

Pepsin is the most potent gastric juice enzyme produced by the cells of the stomach, and it is responsible for transforming proteins into peptides, thus allowing for their absorption.^8,13^ Pepsin is most active between pH is 2.0 and 3.0,^25^ becoming inactive at pH >4.0.^20^ Pepsin was used in this study, together with HCl solution, in the proportion necessary to achieve a final pH of 2.1 to maintain pepsin in its most active pH, and we observed that it produced mild demineralisation in enamel.

The absence of in vitro studies that aim to simulate the duodenal contents has hindered the discussion of the results, and it was necessary to perform preliminary studies to determine the methodology and solutions that should be used. However, we found that bile, in combination with HCl, showed increased erosive potential compared to HCl alone. Hence, duodenal reflux can accelerate and intensify the dental erosion caused by gastroesophageal reflux, thus reinforcing the requirement for dentists to be aware of the signs and symptoms of reflux disease because they may be the first to suspect that patients have gastroesophageal or duodenum-gastroesophageal reflux.
